# Effect of Natural Liquid Hydroabsorbents on Ammonia Emission from Liquid Nitrogen Fertilizers and Plant Growth of Maize (*Zea Mays* L.) under Drought Conditions

**DOI:** 10.3390/plants12040728

**Published:** 2023-02-07

**Authors:** Tomáš Kriška, Petr Škarpa, Jiří Antošovský

**Affiliations:** Department of Agrochemistry, Soil Science, Microbiology and Plant Nutrition, Faculty of AgriScience, Mendel University in Brno, Zemědělská 1, 61300 Brno, Czech Republic

**Keywords:** urea ammonium nitrate, ammonium volatilization, maize, chlorophyll content, root electrical capacitance, plant weight, fertilizer nitrogen recovery

## Abstract

The use of mineral nitrogen (N) fertilizers is associated with significant nitrogen loss through the volatilization. Ammonia (NH_3_) emissions are common from fertilizers with amide (NH_2_) and ammonium (NH_4_) nitrogen forms applied to the soil surface without incorporation. The objective of the laboratory and greenhouse pot experiments was to verify the hypothesis that liquid mineral fertilizers and fertilizer solutions containing N-NH_2_ and N-NH_4_ applied to the soil surface in combination with natural hydroabsorbents (NHAs) will reduce the volatilization of nitrogen. The effect of NHAs addition to urea ammonium nitrate (UAN) fertilizer and urea, ammonium nitrate (AN) and ammonium sulphate (AS) solutions was evaluated in a laboratory experiment. The effect of the two types of NHAs (acidic and neutral) was compared with the control (UAN) and its mixture with the commercially used urease inhibitor N-(n-butyl) thiophosphoric triamide (NBPT). The proportion of volatilized NH_3_ of the total N from the examined fertilizers applied to the soil surface was determined by the titration method. Subsequently, the effect of fertilization with UAN and its mixture with NHAs and NBPT on the growth of maize under the drought conditions was verified in a greenhouse pot experiment. While the addition of NBPT resulted in a reduction of NH_3_ emission for the fertilizers containing NH_2_ (UAN, urea solution), a decrease in volatilization after the addition of both acidic and neutral NHA was observed especially for UAN. A reduction in ammonia emission was also observed for AS after the addition of acidic NHA. The addition of both NHAs and NBPT to UAN increased the utilization of nitrogen from the applied fertilizer, which was reflected by an increase in chlorophyll content and increased CO_2_ assimilation by maize plants grown under the drought stress. UAN fertilizer combined with acidic NHA and NBPT significantly increased aboveground biomass production and root system capacity of maize. Significant increases in UAN nitrogen recovery were observed for all examined additives (UI and both types of NHAs). In addition to the known effects of hydroabsorbents, especially their influence on soil physical and biological properties and soil water retention, the effect of NHAs application in combination with UAN and AS solutions on the reduction of gaseous N loss, maize plant growth and fertilizer nitrogen recovery was found.

## 1. Introduction

Plant nutrition is an important intensification factor in crop production. The major nutrient is nitrogen. The utilization of nitrogen fertilizers accounts for more than half of each other fertilizers combined [1]. Nitrogen is characterized by high mobility in the environment, which is also associated with possible significant loss up to 70% [2]. The percentage of nitrogen loss is mainly affected by the term of application, method of fertilization, incorporation of fertilizers and soil and weather conditions [3]. The important factor determining the efficiency of nitrogen fertilization is a form of nitrogen contained in applied fertilizer. Worldwide, urea is the most widely used in comparison with other nitrogen fertilizers [1]. Urea is usually applied as a solid (granulated), solution or, for certain crops, a foliar spray. It can also be a precursor of another mineral fertilizers containing another form of nitrogen, such as liquid fertilizer urea ammonium nitrate (UAN). UAN has been widely used in the European Union, the United States, Australia and other countries [4]. According to some authors [5,6,7], UAN is the most effective source of mineral nitrogen compared to urea, calcium ammonium nitrate or anhydrous ammonia, because it provides the maximum crop response and availability of inorganic nitrogen content in the soil. However, the urea fertilizers are characteristic by high risk of nitrogen loss via volatilization, which can reach 20–30% under the normal conditions [8]. Urea applied to the dry soil surface without incorporation undergoes rapid hydrolysis to ammonia form [9], and the nitrogen loss can reach more than 50%, especially during warm weather [10]. Ammonia volatilization presents a significant reduction in nitrogen fertilization efficiency, resulting in economic loss. In addition, it also poses a burden for the atmosphere. Up to 80–90% of the total NH_3_ emissions originate in agriculture, of which 10–20% are directly from mineral fertilizers [11]. Therefore, the aim of many studies was to reduce the loss of N by volatilization after the application of nitrogen fertilizers. The incorporation of applied fertilizers to the soil is the basic requirement that results in volatilization decrease. The risk of volatilization is reduced by 14% with every centimetre of depth; therefore, the deeper incorporation is more optimal [12]. A significant reduction in N volatilization can also occur during rain or after irrigation, when the fertilizers are rapidly dissolved [13]. The efficiency of nitrogen fertilization can be increased by the addition of inhibitors of urease (UIs) [14]. UIs slow down or even stop the hydrolysis process of urea molecules to the ammonia. Currently, the most used UI worldwide is N-(n-butyl) thiophosphoric triamide (NBPT) [15]. The addition of NBPT to the urea fertilizers reduces N loss under the field conditions by up to 88% [16,17,18]. However, the effectiveness of NPBT can be relatively short under the drought conditions with high temperatures [19].

The addition of water-retaining agents to conventional fertilizers can help to increase fertilization efficiency. These agents, which are most often based on the absorbent polymers, are primarily applied to optimise soil moisture conditions necessary to overcome a short-term drought period of stress in a relatively quick, low-cost and technologically manageable way [20]. The positive effect of superabsorbent polymers (SAP) on crop production under the water-stressed conditions is well documented by the results of experiments with maize [21,22,23], oilseed rape [24], potatoes [25], tomatoes [26] or ornamental flowers [27]. Although the main function of SAP is passive water retention and reduction of water evaporation from the soil [28], the current research is focusing on the function of SAP as the carrier capable of controlled release of nutrient for crop nutrition. The ability to sorb aqueous nutrient solutions leads to the considerable protection of these nutrients against loss, which can possibly range between 40 and 70% for nitrogen, 80 and 90% for phosphorus and 50 and 70% for potassium [29]. The more gradual or controlled release of nutrients present an optimal coverage of plant demands during the growing season, which increases the efficiency of nutrient use and reduces the negative impact of fertilization on the environment [30]. The three-dimensional crosslinked structure of polymers, primarily dependent on their composition, contains micropores that allow the diffusion of small molecules such as NH_4_^+^ [31]. In addition to diffusion, nutrients are absorbed into the structure of the polymers, delaying their dissolution in the soil water and possible nutrient losses. The release rate of absorbed nutrients is affected by the diffusion properties of the polymer, the nature of the soil environment, the type of SAP used and the form of nutrient.

The synthetic SAP based on potassium polyacrylate is widely used in today’s agriculture. In addition to these inorganic SAPs, there are also bio-based superabsorbent hydrogels called natural hydroabsorbents (NHAs), formed by crosslinking organic compounds such as carrageenan and alginates; modified cellulose; oxidized starch, gelatine and other proteins [32,33,34,35]; crosslinked cellulose acetate [36] and their copolymers with synthetic polymers [37]. The production of biopolymers from bioplastics such as polyhydroxybutyrate or polylactic acid is also a point of interest of the current research [38]. The bio-based natural hydroabsorbent is more degradable in the soil and does not pose such environmental risks in comparison with the conventional inorganic SAPs. While the effect of solid synthetic and natural hydroabsorbents has been documented in several studies, sources evaluating the effect of liquid forms of hydroabsorbents in agriculture are not available [39].

The aim of this presented study was to verify the effect of the addition of neutral and acidic liquid NHAs to the different types of fertilizers on the volatilization of ammonia from liquid N-fertilizers under the laboratory experiment in comparison with the effect of commonly used UI. Subsequently, the effect of fertilization with UAN with acidic or neutral NHAs and with UI in comparison with conventional UAN on the growth and biomass production of maize grown under the drought conditions was investigated in the greenhouse pot experiment. The main hypothesis of the present work is that the addition of NHAs to nitrogen fertilizers applied to the soil surface will reduce ammonia emission. In addition, UAN fertilizer applied in NHA mixture will improve the growth of plants grown under the drought conditions.

## 2. Results and Discussion

### 2.1. The Laboratory Experiment

The application of liquid nitrogen fertilizers with ammonium or amide forms to the soil surface presents a significant risk of ammonia volatilization into the atmosphere. The potential risk of ammonia release increases if the fertilizers are not incorporated to the soil after application. Another factor increasing the volatilization are the drought periods without precipitations [40,41]. As a result, the direct loss of nitrogen used for crop fertilization under the conventional farming systems can range from 10 to 78%, while urea alone can release on average up to 40% of N into the atmosphere within a few days after the application [42,43].

**Figure 1:**
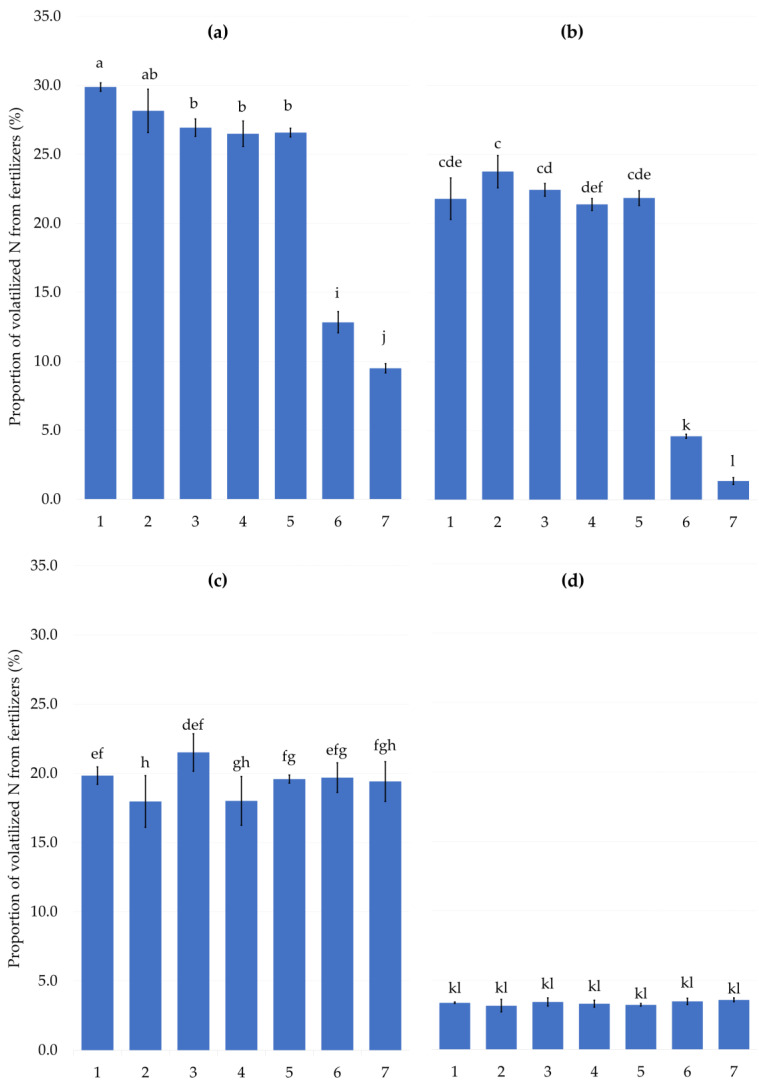
Proportion (%) of volatilized nitrogen from fertilizers (**a**) UAN, (**b**) urea solution, (**c**) solution of AS and (**d**) solution of AN. Experimental treatments: (1) fertilizer without additive, (2) fertilizer + acid NHA (100:1), (3) fertilizer + neutral NHA (100:1), (4) fertilizer + acid NHA (100:2), (5) fertilizer + neutral NHA (100:2), (6) fertilizer + NBPT and (7) fertilizer + StabilureN 30. The columns represent the mean (*n* = 4); error bars present the mean standard deviation. The same letters at the top of the columns describe no statistically significant differences between the treatments (Tukey’s test, *p* ≤ 0.05).

The amount of volatilized nitrogen from examined treatments is presented in Figure 1. The highest statistically significant proportion of ammonia released by volatilization from different fertilizers was recorded for the fertilizer UAN (Figure 1a). The control variant (UAN without any additives) released almost 30% of total applied dose of nitrogen after 5 days, right after the experiment was ended. This corresponds with a several studies, which presents UAN applied only to the soil surface as a significant source of ammonia [44,45]. A relatively large amount of N contained in UAN is susceptible to the volatilization, as the fertilizer contains ½ of nitrogen in urea and ¼ in ammonia form. Turner et al. [46] presented the emission factor as a share of volatilized N in the total applied N dose in UAN. According to their study, the emission factor expressing the proportion of volatilized N in the total applied N rate in the UAN fertilizer can range between 1.8 and 12%. The amount of volatilized ammonia from total N content from SA solution (Figure 1c) was 19.8%, which was very similar to the emission of ammonia from urea solution (21.8%, Figure 1b). The proportion of volatilized ammonia from the total dose of N applied in ammonium nitrate solution was minimal (3.4%), as it is presented in Figure 1d. Nitrogen sources such as ammonium sulphate and ammonium nitrate are not subject to NH_3_ volatilization losses in acid soils [15]. The study performed by Gezgin and Bayrakll [47] described the low volatilization of ammonia from AN, between 4.4 and 6.4%. The proportion of losses are depending on the compounds (phosphor-gypsum and by-product pyrite) and their levels added to the N fertilizers (AN and AS), while the addition of these compounds increased the losses of ammonia compared to the unamended control treatment in their study. The application of ammonium nitrate (AN) to the soil surface, as a fertilizer with low ammonia volatilization potential, was also recommended by Triplett and Dick [40]. Based on the results of our laboratory experiment, fertilizers can be ranked according to their potential risk of volatilization as follows: UAN > urea solution ≥ solution of AS > solution of AN.

The volatilization of ammonia from urea-containing fertilizers can be reduced by the addition of a urease inhibitor. Currently, the most used UI worldwide is NBPT [15]. The NBPT inhibitor was applied in combination with all tested liquid fertilizers, specifically in treatment 6 (lab-prepared NBPT) and treatment 7 (commonly used StabilureN 30). The significant effect of UIs on the reduction of volatilization is in our results evident for UAN fertilizer and urea solution (Figure 1a,b), i.e., fertilizers containing the amide form of nitrogen. The average emission of N measured on treatments 6 and 7 was more than seven times lower compared to the control urea without any addition (Figure 1b). The proportion of ammonia loss from untreated liquid fertilizer containing three effective form of nitrogen (UAN, Figure 1a) was 29.9%, the addition of UIs reduced the ammonia loss to 12.8% (treatment 6) and 9.5% (treatment 7), respectively. The effect of UIs on the reduction of ammonia volatilization from urea fertilizer are well documented by several studies [48,49,50,51,52]. The effect of UI on N volatilization from fertilizers without amide N (AS and AN) was logically not observed (Figure 1c,d). However, the level of volatilized nitrogen varied significantly between these fertilizer solutions. The proportion of volatilized N from AS fertilizer ranged between 18 and 21.5%, while the ammonia from the AN solution was hardly lost. This corresponds with the results of many studies [53,54,55]. The highest proportion of volatilized nitrogen in study of Liu et al. [53] was recorded for ammonium sulphate applied under the drought conditions (20% of field capacity) at 20 °C. Under these conditions, the percentage of N loss was 25.7% of the total applied N, whereas the N loss from NH_4_NO_3_ was roughly half. The emissions of ammonia from nitrogen fertilizers are strongly affected by soil acidity. He et al. [56] described minimal ammonia volatilization at the initial soil pH of 3.5, which increased rapidly with increasing the pH up to 8.5. Correa et al. [55] also presented a lower emission of NH_3_ from AS fertilizer compared to the AN under the field experiment conditions. This result can be explained by the physiologically acidic reaction of the fertilizer, which makes the NH_4_^+^ ions stable. A similar loss of ammonia from AN (0.7%) in comparison with AS (1.0%) was described from a field experiment with maize performed by Souza et al. [54].

The effect of NHAs addition on N volatilization was specific to the fertilizer used, especially the form of nitrogen contained in the respective fertilizers. A significant reduction of ammonia emission was observed after the addition of NHAs (Figure 1a), especially after the treatments 3, 5 (both doses of neutral NHAs) and 4 (higher dose of acidic NHA). The loss of ammonia was reduced from 29.9 (Treatment 1) to 26.9–26.5% by the addition of NHAs to the fertilizers. Such results can be explained by the ability of polymers to bind the ammonium ion and thus directly protect them from volatilization. The important factor is also the effect of absorbent polymers on the soil environment. The published results of Parvathy et al. [57] and Hou et al. [25] proved that the application of hydroabsorbents significantly improved the soil structure, reduced bulk density, increased the soil porosity and water–nutrient holding capacity. According to their composition, the NHAs used in the experiment can be characterized as a group of soil substances with the primary effect of cation sorption, similar to the well-described sorption of clay minerals [58] and organic sorbents [59]. Therefore, the protection of ammonia from volatilization was probably possible due to the binding of NH_4_^+^ to the structure of NHA. The absorbent polymers are also characteristic with a positive effect on soil microorganisms. The addition of hydrogels with different material origins to the soil increases the soil microbial activity [60]. The research of Oksińska et al. [61] proved that the polyacrylate hydrogels have been actively colonized by the soil microbes as a source of carbon and energy. Parvathy et al. [57] described the increase in number of fungi and bacteria in the soil after the addition of natural starch-based hydrogels. An increase in the soil microbial activity is a prerequisite for an increase in biological nutrient sorption, which results in a reduction of soil nitrogen loss. The reduction of volatilization from UAN with the addition of acidic NHA (which significantly acidified the fertilizer) can also be explained by the mentioned effect of the environmental acidity on the soil conversion of NH_4_^+^. The effect of acidic NHA on the emission of NH_3_ in our experiment was also observed in treatments 2 and 4 in AS solution (Figure 1c). A significant reduction from 19.8 to 18.0% was observed in treatment 2 with the higher dose of acidic NHA. This result is probably related to the effect of the solution and soil acidity on the ammonia volatilization from the applied fertilizers. The acidity of the mixtures of the tested fertilizers with addition of NHAs is given in the Table 1. It is evident from this table that the effect of acidic NHA on the pH of the fertilizers (solutions) was significant. The study by Powlson and Dawson [62] showed that the NH_3_ loss from AS fertilizer was under the 5% in the soils with the pH_H_2_O_ < 7.0. On the contrary, the NH_3_ loss in the soils with pH_H2O_ > 7,0 reached up to 20–40%. Similar results are also published by Martens and Bremner [63]. The soil used in our experiment was characteristic with pH_CaCl2_ 6.09. Therefore, the addition of acidic NHA to the AS solution probably contributed to the acidification of the soil environment and, thus, to the reduction of N volatilization.

The described effect of NHAs addition demonstrated for UAN and AS fertilizers was not confirmed for urea and AN. The level of volatilization in case of AN solutions was very low; therefore, the effect NHAs could be hardly observed. The effect of NHAs addition to the urea fertilizer was not expected.

### 2.2. The Greenhouse Experiment

**Figure 2:**
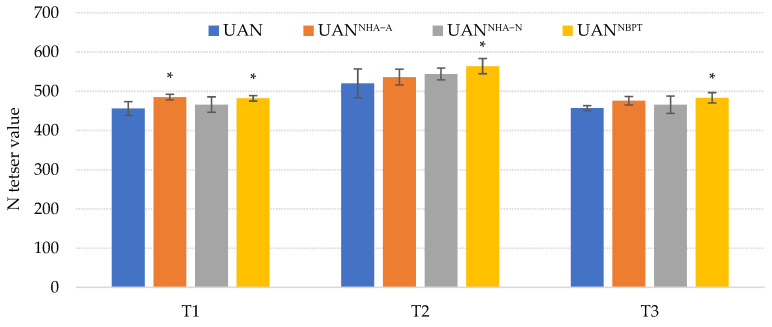
The effect of the UAN fertilizer with NHAs and NBPT on chlorophyll contents (N-tester value). The columns represent the mean (*n* = 12); error bars present the mean standard deviation. The mean values marked with asterisk are significantly different (*p* ≤ 0.05) from the treatment UAN by Tukey’s test (each of the terms was statistically evaluated separately).

The effect of NHAs and UIs applied in combination with UAN fertilizer were observed in the chlorophyll content in maize leaves (N-tester value). The N-tester values was significantly increased in each term (T1–T3) on the treatment with UAN^NBPT^ (Figure 2). This corresponds with the ability of the inhibitor to reduce the ammonia loss (Figure 1), which is reflected in an enhanced N-fertilization effect. One of the many common responses of plants fertilized with urea supplemented with UI is an increase in the leaf chlorophyll content [64,65,66,67]. In agreement with our results, Liu et al. [68] reported a significant increase of the leaf chlorophyll in maize plants after the surface applied or knifed-in UAN treated with NBPT. A relative increase in the chlorophyll content in maize leaves fertilized with UAN^NHA-A^ was detected in each term, averaging a 4.5% increase compared to the control treatment UAN. The effect of acidic NHA addition can be explained by the increased availability of nitrogen supplied to plants by the fertilizer, caused by the acidification of the soil. The acidification of soil proved to have a significant effect on the abiotic NH_3_ volatilization [69,70,71]. The effect of neutral NHA addition on leaf chlorophyll content was not observed. Maize fertilized with UAN in combination with neutral NHA showed almost the same development of the N-tester value as the UAN variant.

**Figure 3:**
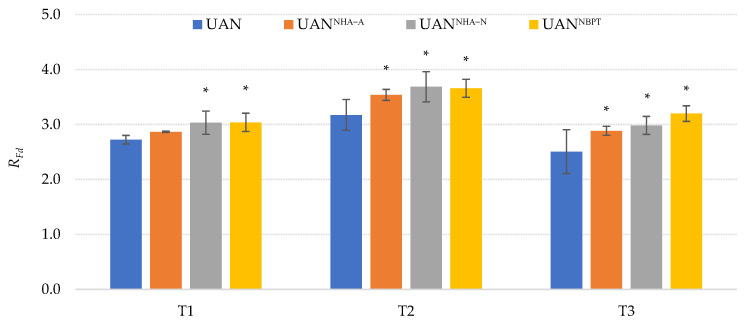
The effect of the UAN fertilizer with NHAs and NBPT on chlorophyll fluorescence decrease ratio (*R_Fd_*). The columns represent the mean (*n* = 12); error bars present the mean standard deviation. The mean values marked with asterisk are significantly different (*p* ≤ 0.05) from the treatment UAN by Tukey’s test (each of the terms was statistically evaluated separately).

The application of the NHAs and UIs to the UAN fertilizer also affected the fluorescence decrease ratio (*R_Fd_*), which is directly proportional to the rate of CO_2_ assimilation. The reduction of nitrogen losses via volatilization due to the addition of NHAs or NBPT described in our results probably led to an improvement in the nutritional status of the plants, which physiologically resulted in improved CO_2_ assimilation by the plants. A study by Dewi et al. [66] reported that the plants treated with urea containing urease inhibitors (NBPT and NPPT) showed a higher chlorophyll content and transpiration rate. These factors influenced the ability of the plant to photosynthesize. The significantly highest *R_Fd_* values were recorded on treatment UAN^NBPT^ and UAN^NHA-N^ (Figure 3) in each term (T1–T3). The average *R_Fd_* value was higher by 17.8, respectively, 15.5% in comparison with UAN. The plants were grown under the limited watering regime. Taking this into account, it is possible that the positive effect on improving assimilation could also be explained by the ability of NHAs to retain water. This result corresponds with the findings of several studies evaluating the effect of superabsorbent polymer on the growth and production of maize grown under the drought conditions [72,73,74].

**Figure 4:**
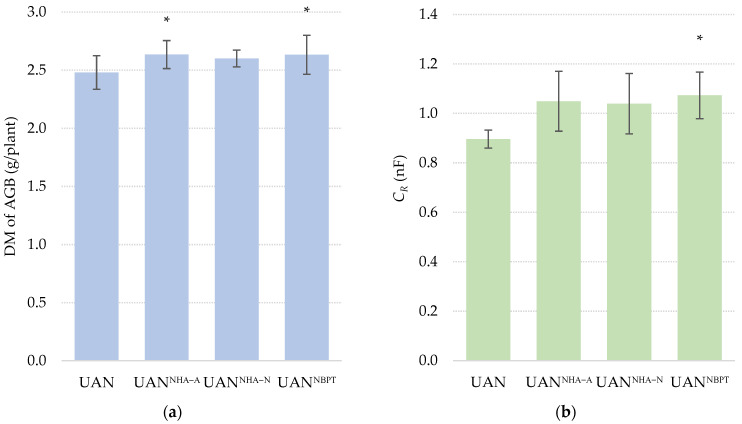
The effect of the UAN fertilizer with NHAs and NBPT on (**a**) weight of DM of AGB, and (**b**) root electrical capacitance of maize (*C_R_*). The columns represent the mean (*n* = 12); error bars present the mean standard deviation. The mean values marked with asterisk are significantly different (*p* ≤ 0.05) from the treatment UAN Tukey’s test.

The obvious increase in production of maize AGB (Figure 4) can be easily explained by the positive effect of NHAs, especially on retaining water for plants under the drought conditions [75] and reduction of N volatilization. The weight of maize AGB observed after the fertilization with UAN enhanced with acidic NHA was increased by 6% compared to the control treatment (UAN). This result is similar to the effect of NBPT addition to UAN fertilizer presented by Grant et al. [76]. UAN treated with NBPT or with combination of urease and nitrification inhibitors resulted in a 16.5–16.6% higher maize yield in comparison with the untreated UAN when applied to a surface [68]. The reduction of NH_3_ volatilization and increase in yield of wheat grain fertilized by UAN with NPBT was described by Nikolajsen et al. [45]. In addition to the above benefit, UI applied in combination with UAN near the seed row of canola significantly decreased seedling damage [76]. The weight of maize AGB was in correlation with the size of the maize root system (r = 0.835, *p* ˂ 0.001), measured as the root capacitance (*C_R_*). This parameter is closely related to the length and total mass of the roots [77]. The most significant increase in root system of maize was observed after the fertilization with UAN an NBPT. Zanin et al. [78] proved that the presence of NBPT in urea fertilizer reduced the ability of plants to use this form of nitrogen as a source nutrient. However, UAN fertilizer combines several forms of nitrogen not affected by the presence of UI. Therefore, it provides plants with a constant supply of nitrogen during the growing season [79]. The results presented in our experiment are therefore in correlation with several studies describing root growth promotion by the addition of NBPT to urea-containing fertilizers [66,76,80].

Some studies reported that UAN is the most efficient nitrogen source compared to other N-fertilizers (urea and calcium ammonium nitrate), because it provides the maximum crop response and availability of soil mineral N content [5,81]. Less residual and surplus nitrogen in the soil was found after UAN fertilization compared to urea fertilization of wheat [82] and maize [7]. Ren et al. [83] reported that the application of UAN without additives was beneficial for the dry matter accumulation of maize, and compared with urea treatment, the recovery rate of nitrogen for UAN treatment was increased by 31.2%. In our experiment, the significantly highest nitrogen content in AGB was observed in maize fertilized with UAN^NBPT^_._ This treatment also provided the significantly highest N uptake by DM of AGB and fertilizer nitrogen recovery (*FN_RE_*). The *FN_RE_* increase was 2.8% compared to the UAN treatment (Table 2). The fertilizers containing N-NH_2_ in combination with NBPT increased nitrogen use efficiency of maize [84,85], wheat [86,87] and others crops [88]. A significant *FN_RE_* increase was also observed in maize fertilized with both UAN^NHA-A^ and UAN^NHA-N^. The natural hydroabsorbents increased the water availability to maize plants under the drought conditions, leading to an increase in the availability of applied nitrogen. Maize suffers from several production constraints, of which limited water availability and nitrogen deficiency are the most restricting factors, frequently occurring together [89]. Rimski-Korsakov et al. [90] reported that, while maize grown under the drought conditions uptakes N from fertilizer independently of the N rate, the nitrogen uptake increased with the optimal water regime. The positive effect of hydroabsorbents addition to fertilizers on the nitrogen uptake by plants *Pinus massoniana* was also described by Mao et al. [91]. Mixture compound fertilizer and superabsorbent polymer application significantly increased the nitrogen content in the leaves, stems and roots of the seedlings.

The majority of published studies on the use of hydroabsorbents in crop production have only tested their effect on the physicochemical properties and water retention ability of the soils with their consequent impact on the yield and plant quality. The effect of natural hydroabsorbent on the emission of N and the impact of their co-application with liquid N fertilizers on the plant growth has been currently researched in a very limited way. However, the liquid NHAs have a high potential for use in plant nutrition due to their composition. The reduction of ammonia emissions due to the addition of both acidic and neutral NHA was observed in our experiment, especially for UAN fertilizer. The liquid natural hydroabsorbents are combining the ability to increase water retention and to improve the utilization of applied nitrogen by plants, which makes them suitable nutritional supplements for plants grown, especially in arid regions. The further research will be aimed at verifying the effect of mixtures of NHA with liquid N fertilizers, especially UAN, under field conditions.

## 3. Materials and Methods

### 3.1. The Laboratory Experiment

The main aim of this experiment was to examine the effect of NHAs on the ammonia volatilization from selected nitrogen fertilizers. The experiment was conducted under the laboratory conditions at the Department of Agrochemistry, Soil Science, Microbiology and Plant Nutrition, Faculty of AgriSciences, Mendel University in Brno (49°12′36.94″ N and 16°36′49.95″ E).

#### 3.1.1. Materials

The effect of NHAs and UIs addition on ammonia volatilization was examined from liquid urea ammonium nitrate (UAN; 19.5% N-NH_2_, 9.75% N-NH_4_^+^ and 9.75% N-NO_3_^−^; ACHP, Slavkov Inc., Slavkov u Brna, Czech Republic) and solutions of fertilizers: liquid urea solution (urea; 46% N-NH_2_; Duslo Inc., Šal’a, Slovakia), liquid solution of ammonium sulphate (AS; 21% N-NH_4_^+^, CAS: 7783-20-2, Sigma Aldrich, St. Louis, MO, USA) and liquid solution of ammonium nitrate (AN; 17.2% N-NH_4_^+^ and 17.2% N-NO_3_*^−^;* CAS: 6484-52-2, Sigma Aldrich, St. Louis, MO, USA). The liquid NHAs were used for the experiment. The liquid NHAs were a dispersion of modified polysaccharide hydrogel microparticles in an aqueous solution of commercial non-ionic surfactants (Aqueduct), natural hygroscopic compound and natural biostimulant, with the pH adjusted to 6.7 (neutral NHA, NHA-N) and 2.6 (acid NHA, NHA-A). The effects of NHAs were also compared with lab-prepared UI N-(n-butyl)thiophosphoric triamide (NBPT) made according to Santos et al. [92] and with commercially used UI StabilureN 30 (AGRA GROUP Inc., Střelské Hoštice, Czech Republic).

#### 3.1.2. Experimental Design and Measurements

The examined fertilizer (UAN) and fertilizer solutions (urea, AS and AN) were applied to the experimental containers in the dose of nitrogen described in Table 3. UAN fertilizer was applied in an undiluted form, and the examined solutions were prepared as follows: urea solution (42.4 g urea dissolved in 100 mL distilled water), AS solution (62 g AS dissolved in 100 mL distilled water) and AN solution (56.6 g AN dissolved in 100 mL distilled water). The fertilization was performed by micropipette on the soil surface. The applications of the examined treatments are described in Table 4; each treatment (1–7) was repeated for UAN and every fertilizer solution (urea, AS and AN). Each treatment was established in 4 repetitions (hermetic chambers).

The basic characteristics of the soil used in the experiment are given in Table 5 [93]. Twenty grams of this soil with were weighted into the 4 cm of diameter plastic containers (50 mL volume).

**Figure 5:**
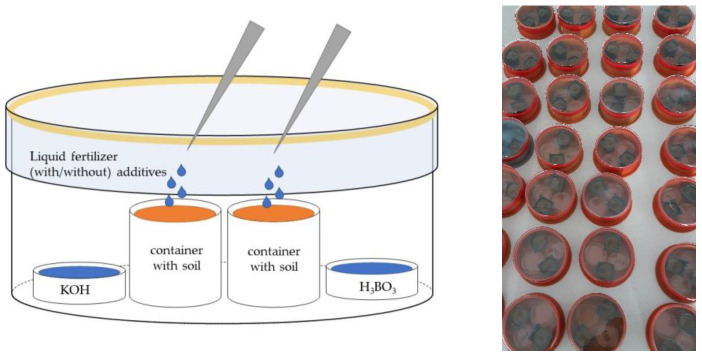
Hermetic chamber to quantify NH_3_ volatilization from fertilized surface soil.

Two plastic containers with soil were placed into the hermetic chamber together with a petri dish containing 5 mL 4% boric acid (B0394/CAS: 10043-35-3; Sigma Aldrich, St. Louis, MO, USA) served as a sorbent for ammonia and a petri dish containing 4 mL 5% KOH (306568/CAS: 1310-58-3; Sigma Aldrich, St. Louis, MO, USA) for capturing CO_2_. The examined fertilizers solely or in combination with NHAs and UIs were then applied by micropipette on the soil surface of each container. Both types of NHA (acidic and neutral), lab-prepared NBPT solution and commercial StabilureN 30 were mixed into the fertilizers 10 min before their application at the ratios shown in Table 4. The dosage of NBPT in lab-made UI (NBPT) and commercial UI (StabilureN) applied to the liquid fertilizers were identical. The chambers were sealed by a petri dish with Ramsay’s grease immediately after the application of fertilizers (Figure 5). The hermetic chamber was left for 120 h (5 days) in the growth box (PlantMaster, CLF Plant Climatics GmbH, Wertingen, Germany) under precisely controlled temperature and light conditions (21 °C, 24 h night). The experiment ended after 5 days, as the content of the boric acid in the petri dish was quantitatively transferred using distilled water to a titration flask. Subsequently, its content was titrated with 0.02 M H_2_SO_4_ (339741/CAS: 7664-93-9; Sigma Aldrich, St. Louis, MO, USA) until the equivalence point was reached and the colour changed on the Tashiro indicator (0.75 g/L methyl red sodium salt + 0.375 g/L methylene blue in ethanol 50% (*v*/*v*), denatured).

The amount of volatilized ammonia was calculated according to the consumed sulphuric acid solution, as it is described in Equation (1).

Volatilized N-NH_3_ (µg) = V ∗ f ∗ 0.56 (1)

In Equation (1), V = consumption of H_2_SO_4_ (mL); f = purity factor of H_2_SO_4_ solution.

The amount of N-NH_3_ was subsequently expressed as a % of the total applied amount of N in fertilizers (their solutions).

### 3.2. The Greenhouse Experiment

The pot experiment with maize (*Zea mays* L.) was established after the completion of the lab experiment. The aim of this vegetation experiment was to examine the effect of UAN fertilizer in combination with NHAs and UIs on the growth and biomass production of maize in the greenhouse.

#### 3.2.1. Materials and Experimental Design

The greenhouse pot experiment under the controlled conditions (length: 12 h light, 12 h night; humidity: 60% day, 80% night; temperature: 25 °C day, 17 °C night) was carried out in the growth box (PlantMaster, CLF Plant Climatics GmbH, Wertingen, Germany) in the Biotechnological house at Mendel University in Brno. Plants were grown in 2 L plastic pots with 1500 g of arable soil. The basic characteristics of soil used in the greenhouse experiment are described in Table 5, as the soil was identical as in the first experiment. Four seeds of maize, variety SY ORPHEUS (Syngenta Czech Ltd., Prague, Czech Republic), were sown in each pot. The plants were thinned down in each pot to a final count of three plants right after emergence.

The fertilizers were applied to the soil surface immediately after the sowing; the examined treatments are described in Table 6. The dose of applied nitrogen was 0.4 g N/pot (1.33 mL UAN per pot), and it was identical for each treatment. Each treatment was established in four repetitions, and the vegetation pots were arranged randomly in the growth box.

The first watering was done 5 days after the fertilization. The maize was exposed to drought during the duration of the experiment (30% water-holding capacity). The watering regime was maintained on a gravimetric basis [94].

#### 3.2.2. Measurement of Maize Growth Parameters

**Figure 6:**
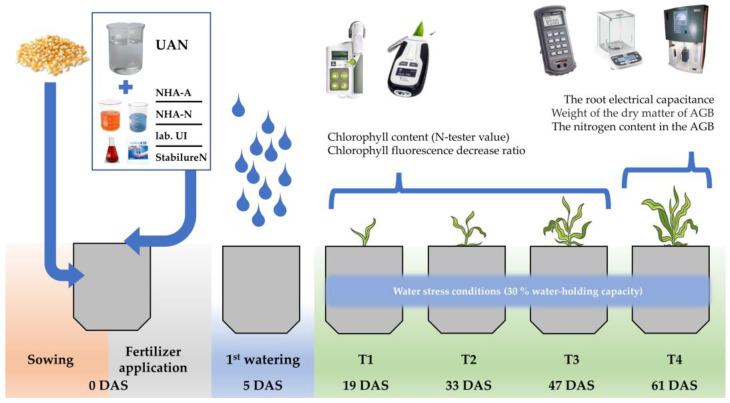
Timetable of the greenhouse pot experiment. DAS—day after sowing.

The effect of examined treatments on the growth of maize plants was evaluated by the measurements of the selected growth parameters, specifically the content of chlorophyll (N-tester value) and chlorophyll fluorescence decrease ratio (Table 7). The measurements of these parameters were carried out in 3 terms at an interval of 14 days (T1–T3). The first measurement took place 14 days after the watering (Figure 6). The greenhouse pot experiment was terminated by measuring of the root electrical capacitance and weight of dry matter of the aboveground biomass of a plant (T4).

Chlorophyll content (N-tester value) was determined by hand-held optical chlorophyll meter Yara N-Tester^TM^ (Yara International ASA, Oslo, Norway). The chlorophyll fluorescence decrease ratio (*R_Fd_*) of leaves was measured with the fluorometer PAR-FluorPen FP 110-LM/S (Photon Systems Instruments, Drásov, Czech Republic) using the measurement protocols according to Škarpa et al. [97]. The software FluorPen 1.1 was used for the analysis of the measured data. Measurements of the chlorophyll content and *R_Fd_* (Figure 2) were carried out at each term (T1–T3) and each pot (each treatment with 4 repetitions and a total of 3 plants = 12 measurements). The chlorophyll content and *R_Fd_* were determined from the middle of the 2nd leaf (T1), 3rd leaf (T2) and 4th leaf (T3), with the same leaves for all treatments.

The root electrical capacitance (*C_R_*) was measured using a portable hand digital multi-metre VOLTCRAFT LCR 4080 (Conrad Electronic GmbH, Wels, Austria) at the end of the experiment (T4). *C_R_* was determined in nano-farads (nF), and a frequency signal of 1 kHz was used. Before determining *C_R_*, the pots were irrigated to a level of 100% of the full water capacity. The 1st electrode (ground), a 20 cm long stainless-steel rod, was inserted vertically to a 15 cm depth in the soil at a distance of 5 cm from the plant stem. The 2nd electrode (plant), an aluminium clamp, was placed on the plant stem at a height of 2 cm above the soil surface. The measurement of *C_R_* was performed for each treatment in 12 repetitions.

After *C_R_* determination, the plant aboveground biomass (AGB) was cut at the base of the stem, and the mass of the vegetative matter was harvested. The samples of plant mass were dried in the drying oven with air circulation (Venticell 222 ECO line, MMM Medcenter Einrichtungen GmbH, Planegg, Germany) to the constant weight at a temperature of 60 °C. The weight of the dry matter (DM) of AGB was determined using the digital scale Kern ADB 200-4 (KERN and Sohn GmbH, Balingen, Germany). The determination of plant AGB (g/plant) was performed for each treatment in 4 repetitions (each repetition is the average weight of 3 plants per pot). The nitrogen content in the AGB of maize plants was determined according to the methodology by Zbíral et al. [98]. The Kjeldahl method was used for the determination of nitrogen in plants (Kjeltec 2300 device, Foss, Hillerød, Denmark).

The fertilizer nitrogen recovery efficiency of maize production (AGB) was calculated using the following equations:

Accumulated N in the AGB of maize (N uptake): AN (mg/pot)

AN = (DM ÷ 100) × N × 1000(2)

In Equation (2), N is the N content in the AGB of maize (% of DM), and DM is the total dry matter (g/pot).

Fertilizer nitrogen recovery (*FN_RE_*, %) in the AGB of maize was determined. N utilization refers to the N recovered from fertilizer applied to pots:

FN_RE_ = (AN ÷ ANA) × 100 (3)

In Equation (3), ANA is the amount of N applied in fertilizer (mg/pot).

### 3.3. Statistical Data Analysis

The effect of examined treatments on the monitored parameters was statistically evaluated in both experiments. Statistical evaluation was performed by Statistica 13 CZ software [99] (TIBCO Software, San Jose, CA, USA). The relationship between the treatment’s one-way analysis of variance (ANOVA) and follow-up tests according to Tukey’s at the 95% (*p* < 0.05) level of significance were used, and homogeneity and normality of variances were tested by Levene’s and Shapiro–Wilk tests, respectively (*p* ≤ 0.05 level). The results were expressed as the mean ± standard deviation (SD).

## Figures and Tables

**Table 1:** The effect of NHAs addition on the acidity of liquid fertilizer mixtures.

Tr.	Additive		UAN	AS	Urea	AN
1	-	-	7.70	5.18	8.82	4.58
2	acid NHA	100:1	7.26	3.25	3.74	2.34
3	neutral NHA	100:1	7.60	4.65	5.39	4.35
4	acid NHA	100:2	5.29	3.06	3.52	2.18
5	neutral NHA	100:2	7.58	4.60	5.30	4.38

NHA—natural hydroabsorbent; UAN—urea ammonium nitrate; AS—solution of ammonium sulphate; Urea—urea solution; AN—solution of ammonium nitrate.

**Table 2:** The effect of the UAN fertilizer with NHAs and NBPT on the content of N in AGB of maize, nitrogen uptake (AN) and fertilizer N recovery (*FN_RE_*).

Treatment	N Content % DM	AN (N Uptake) mg/pot	*FN_RE_* %
UAN	1.17 ^b^ ± 0.04	87.0 ^b^ ± 5.0	21.8 ^b^ ± 1.3
UAN^NHA-A^	1.19 ^ab^ ± 0.03	95.0 ^a^ ± 3.2	23.8 ^a^ ± 0.8
UAN^NHA-N^	1.21 ^ab^ ± 0.03	95.2 ^a^ ± 4.0	23.8 ^a^ ± 1.0
UAN^NBPT^	1.25 ^b^ ± 0.07	98.3 ^a^ ± 6.6	24.6 ^a^ ± 1.6

The mean values sharing the same superscript are not significantly different from each other (*p* ≤ 0.05), according to Tukey’s test. The values represent the mean (*n* = 12) ± standard deviation. UAN: urea ammonium nitrate; NHA-A: acid natural hydroabsorbent; NHA-N: neutral natural hydroabsorbent; NBPT: N-(n-Butyl)thiophosphoric triamide.

**Table 3:** Application dose of nitrogen (µg/hermetic chambers).

Fertilizer	N-NH_4_^+^	N-NO_3_*^−^*	N-NH_2_	N Total
UAN	19.5	19.5	39.0	78.0
Urea solution	0.0	0.0	39.0	39.0
Ammonium sulphate solution	19.5	0.0	0.0	19.5
Ammonium nitrate solution	19.5	19.5	0.0	39.0

**Table 4:** Examined treatments repeated for UAN and each fertilizer solution.

Treatment	Additives	Fertilizer (Fertilizer Solution): Additive Ratio
1	-	-
2	acid NHA	100:1
3	neutral NHA	100:1
4	acid NHA	100:2
5	neutral NHA	100:2
6	NBPT	100:0.3
7	StabilureN 30	100:0.1

**Table 5:** Chemical composition of soil used in the lab experiment.

Soil Parameter	Value
pH_CaCl2_	6.09
Soil oxidizable carbon (C_ox_)	0.80%
Clay	20%
Silt	27%
Sand	53%
Cation exchange capacity	164 mmol/kg
N total	0.19%
N-NH_4_^+^ (K_2_SO_4_)	1.48 mg/kg
N-NO_3_^−^ (K_2_SO_4_)	17.2 mg/kg
P (Mehlich 3)	36.4 mg/kg
K (Mehlich 3)	400 mg/kg
Ca (Mehlich 3)	2720 mg/kg
Mg (Mehlich 3)	214 mg/kg

**Table 6:** Treatments of the greenhouse experiment.

Treatment	Additives	Fertilizer (Fertilizer Solution): Additive Ratio
UAN	-	-
UAN^NHA-A^	acid NHA	100:2
UAN^NHA-N^	neutral NHA	100:2
UAN^NBPT^	NBPT	100:0.3

**Table 7:** Photochemical quenching parameters.

Parameter		Ref.
Chlorophyll fluorescence decrease ratio (*R_Fd_*)	*F_d_*/*F_s_*	[95]
Chlorophyll content (N-tester value)		[96]

*F_d_* means fluorescence decrease from *F_m_* to *F_s_*, *F_m_*: maximal fluorescence from the dark-adapted leaves, *F_s_*: steady-state chlorophyll fluorescence.

## Data Availability

Not applicable.
